# The Value of Cardiopulmonary Exercise Testing in Predicting the Severity of Coronary Artery Disease

**DOI:** 10.3390/jcm11144170

**Published:** 2022-07-18

**Authors:** Wanjun Liu, Xiaolei Liu, Tao Liu, Yang Xie, Xingwei He, Houjuan Zuo, Hesong Zeng

**Affiliations:** 1 Department of Cardiology, Tongji Hospital, Tongji Medical College, Huazhong University of Science and Technology, Wuhan 430030, China; wjliu@tjh.tjmu.edu.cn (W.L.); lxl2015tjh@163.com (X.L.); taoliu_6@126.com (T.L.); xieyang01010@163.com (Y.X.); hexingwei2004@163.com (X.H.); 2 Hubei Key Laboratory of Genetics and Molecular Mechanisms of Cardiological Disorders, Wuhan 430030, China

**Keywords:** cardiopulmonary exercise testing, cardiorespiratory fitness, coronary artery disease, Gensini score, quantitative flow ratio

## Abstract

*Background:* There have been a limited number of quantitative studies on the relationship between coronary artery disease (CAD) and cardiorespiratory fitness (CRF), as measured by cardiopulmonary exercise testing (CPET). Thus, we aimed to investigate the association between CRF and the severity of coronary artery disease from the most comprehensive perspective possible, and to affirm the predictive value of CPET in the severity assessment of CAD. *Methods:* Our study included 280 patients with coronary angiography, who had undergone CPET in Tongji Hospital. The patients’ CRF was measured through their peak oxygen uptake (VO_2_@peak), their oxygen uptake at the anaerobic threshold (VO_2_@AT) and through other parameters of CPET on a bicycle ergometer. The severity of the coronary artery disease was assessed in the following three layers: functionally significant lesions (quantitative flow ratio [QFR] ≤ 0.8), the number of stenotic coronary arteries (SCA, stenosis ≥ 50%) and the Gensini score. The correlation analyses were carried out between the CRF and the severity of the coronary artery disease. A ROC curve was plotted, and the AUC was calculated to distinguish the severe CAD and the non-severe CAD patients, as measured by the QFR, the number of SCA, and the Gensini score. *Results:* The VO_2_@AT and VO_2_@peak were inversely associated with the QFR. The VO_2_@AT, VO_2_@peak and VO_2_/kg@peak were associated with the number of SCA. Meanwhile, the VO_2_@AT, VO_2_/kg@AT, VO_2_@peak and VO_2_/kg@peak were associated with the Gensini score. An ROC analysis proved that a combination of traditional clinical risk factors and the VO_2_@peak/VO_2prediction_ is valuable in predicting CAD severity. *Conclusions:* Our study demonstrated a strong and inverse association between CRF and the severity of CAD. A combination of traditional clinical risk factors and CRF is valuable in predicting CAD severity.

## 1. Introduction

Cardiopulmonary exercise testing (CPET) is a useful and objective assessment of the body’s response to exercise. Compared to traditional tools for evaluating cardiac function, such as the six-minute walk test, the exercise electrocardiography test and the pulmonary function test, CPET possesses many advantages. It can be used to measure a broader range of variables related to cardiorespiratory function by combining exercise physiological data with noninvasive recordings of cardiac function [1]. It provides greater diagnostic accuracy, additional prognostic information, more precise determination of aerobic capacity and a unique insight into the independent and coupled functions of the cardiovascular, respiratory, skeletal and neurophysiologic systems [2,3]. CPET can be used to assess the cardiac functions and prognosis of heart failure patients, as well as to establish the indication of cardiac transplantation in cardiology [4,5]. In the past decade, there has been an exponential increase in evidence regarding the use of CPET in specific patients [5]. Among them, CPET for coronary artery disease (CAD) assessment is an area of growing clinical interest, in which different parameters provide both diagnostic and prognostic insight for evaluation and management [6,7]. Exercise stress testing has traditionally been used to identify obstructive CAD (O-CAD), with the intent of revascularizing the culprit coronary lesions [8]. Furthermore, while objective techniques for quantitative analysis are lacking in the diagnosis of CAD, they are critical for interventional decision making, as there is growing evidence in the literature that revascularization may only be advantageous to individuals with significant degrees of ischemia [9,10]. CPET may have added value in CAD quantification and prognosis since it allows for a comprehensive evaluation of the metabolic changes and the overall cardiopulmonary systems, and its results are objectively expressed in numbers [11,12,13]. The peak oxygen consumption (VO_2_@peak) is assessed by standardized CPET, which is considered the gold standard for assessing CRF, and is strongly associated with cardiovascular mortality. Letnes et al., reported that even among a healthy, low-risk population, the VO_2_@peak was inversely associated with CHD [14]. To provide more evidence, we conducted comprehensive statistical analyses of the correlation between the VO_2_@peak and the coronary artery severity in 280 patients with non-negative coronary angiography.

## 2. Materials and Methods

### 2.1. Study Design and Patients

This was a retrospective study, conducted in Tongji Hospital, Affiliated to Tongji Medical College, Huazhong University of Science and Technology. Ethical approval was granted by the Tongji Hospital Ethics Committee (TJ-IRB20220314). Patients with suspectable coronary artery disease symptoms, who had undergone coronary angiography and CPET at Tongji Hospital were included in this study. Patients with normal coronary vessels (as shown in the coronary angiography), chronic lung disease and insufficient data were excluded. The inclusion criteria were patients with stenotic coronary arteries and CPET. A coronary angiography was performed to confirm the presence of coronary artery disease and to define the severity of the coronary artery lesions before a symptom-limited exercise tolerance test was conducted to assess the exercise capacity of the patients. Tea, coffee, cola drinks, chocolate and smoking were not allowed for 24 h before the evaluation. A total of 280 patients (188 men and 92 women; 55.90 ± 8.65 vs. 58.21 ± 7.17 years) with coronary artery lesions were finally included in our study. All subjects were clinically stable and did not exercise regularly.

### 2.2. Data Collection

Baseline patient parameters, including age, height, body weight and past medical history, were collected on admission, while medication use and exercise testing results were obtained through standardized data collection forms from electronic medical records. To ascertain the symptom data, which were not available from electronic medical records, the researchers directly communicated with patients to ascertain the data on the day of the CPET.

### 2.3. CPET

To monitor the cardiorespiratory status and make a pre-judgement about the severity of the coronary artery lesion by monitoring the electrocardiogram and symptoms (such as chest tightness and chest pain) and through other CPET parameters mentioned below, each patient underwent CPET one to three days before coronary angiography. CPET was performed on a cycle ergometer, with a personalized ramp exercise protocol aimed at achieving peak exercise in approximately 10 min until exhaustion, with continuous measurement and monitoring of oxygen consumption, carbon dioxide production, ventilatory capacity and hemodynamic indices. In short, the patients were instructed to rest for 3 min to allow the gas exchange variables to stabilize, followed by 3 min of freewheel pedaling and then they would reach peak exercise with a gradual increment in workload, targeted to the specific exercise capability of each patient. Another 5 min passed in order to allow the patients to recover. Ventilation and gas exchange were measured using a metabolic cart. Heart rate, full disclosure 12-lead electrocardiogram, blood pressure and pulse oximetry were monitored throughout. Tests were symptom-limited (i.e., fatigue, dyspnea and angina), or were stopped when one of the following criteria was met: achieving respiratory exchange ratio (RER) ≥ 1.15; a hypertensive response to exercise (≥220/110 mmHg); or ≥3 mm ST depression in at least two adjacent leads. The following CPET parameters were measured at rest, at the anaerobic threshold and at peak: oxygen uptake (VO_2_) and RER. The ratios of VO_2_ to body weight were denoted as VO_2_/kg. VO_2_@peak and RER@peak was the average of last 10 s of CPET. AT was identified using a V-slope analysis of VO_2_ and VCO_2_, and it was confirmed by specific trends of ventilatory equivalent for oxygen (VE/VO_2_) and ventilatory equivalent for carbon dioxide (VE/VCO_2_) and by the end-tidal pressure of oxygen and end-tidal pressure of CO_2_.

### 2.4. Coronary Angiography

Left and right coronary angiographic examinations were performed via radial or femoral artery puncture and multi-position projection was performed according to Judkins’ method, by experienced operators. Suspect lesions were diagnosed independently by two interventional physicians, blinded to the other for each patient, and the analysis was performed by a third physician when necessary. The degree of stenosis of the left main trunk, left anterior descending branch, left circumflex branch, and right coronary artery in each patient was assessed and recorded during coronary angiography.

### 2.5. QFR

QFR analysis was performed analogously to previous studies using Medis proprietary software (AngioPlus, Shanghai Pulse Medical Technology Inc., Shanghai, China). Following the image choice, end-diastolic frames were selected, the target segment of the considered vessel was manually determined and the automatically detected vessel contour was checked and corrected when necessary. Flow QFR was obtained by the semiquantitative assessment of contrast dye flow, as allowed by the commercial software used. Flow QFR was used for further analysis. QFR of left anterior descending branch, left circumflex branch, and right coronary artery of each person were obtained from the software independently by two interventional physicians, with a third physician participating in the evaluation when there was disagreement. Final QFR values were computed using 3D-QCA and frame counting (the so-called contrast QFR model).

### 2.6. Gensini Score

The severity of coronary artery disease (CAD) is determined by calculating a modified Gensini score, based on the scoring schema established by Gensini et al. [15]. In brief, 1 point is given if any branch of the main coronary artery, including the left main artery (LM), the left anterior descending (LAD), the left circumflex coronary artery (LCX) and the right coronary artery (RCA) has stenosis reaching 1–25% of the internal lumen diameter. Similarly, 2 is allotted for 26–50% stenosis, 4 for 51–75%, 8 for 76–90%, 16 for 91–99% and 32 for 100% or total occlusion. Depending on the location of the lesion, the single lesion score is multiplied by different coefficient, and the final Gensini total score is calculated (Supplementary Table S1). The Gensini scores were calculated by three independent cardiologists to avoid observation bias and the mean value of the three was selected for the statistical analysis. Severe CAD was defined as the Gensini score ≥ 20 [16].

### 2.7. Assignment and Grouping

(1) QFR assignment and grouping. The enrolled patients were divided into two groups according to QFR (QFR > 0.8 and QFR ≤ 0.8). Those with one or more than one coronary arteries with QFR ≤ 0.8 were assigned 0, while the patients with no coronary artery with QFR ≤ 0.8 were assigned 1.
(2) The patients were divided into three groups, according to the number of stenotic coronary artery (0, 1–2 and 3–4, respectively). Assignment of CPET indices (VO_2_@peak, VO_2_@AT, VO_2_kg@peak and VO_2_@AT). VO_2_@peak, VO_2_@AT, VO_2_kg@peak and VO_2_@AT of the male and female patients were arranged in descending order, respectively, and divided into four equal groups, with values of 1, 2, 3 and 4.
(3) Gensini scores were grouped by quartile. The four groups of males were group 1 (Gensini score ≤ 6.0), group 2 (6.0 < Gensini score ≤ 12.5), group 3 (12.5 < Gensini score ≤ 27.5) and group 4 (Gensini score > 27.5), respectively. The four groups of females were group 1 (Gensini score ≤ 3.0), group 2 (3.0 < Gensini score ≤ 7.5), group 3 (7.5 < Gensini score ≤ 14.5) and group 4 (Gensini score > 14.5), respectively. Then the enrolled subjects were divided into the four groups according to their Gensini score.

### 2.8. Statistical Analysis

Statistical analyses were performed using SPSS software (Version 26.0, SPSS, USA). Participants were grouped by gender to eliminate potential gender differences. For baseline data, continuous variables were expressed as mean and standard deviation, while categorical data were expressed as proportions. The distribution of all CPET parameters were tested for normality by the Shapiro–Wilk normality test. Due to the non-normality distribution, CPET parameters were expressed as median and quartiles. Comparisons of CPET parameters between two groups were performed by Mann–Whitney non-parameter test and comparisons among more than two groups were performed using Kruskal-Wallis non-parameter test. The Spearman correlation test was used to assess correlation between the Gensini score and the CPET parameters, as well as the QFR and CPET parameters. The Kendall’s tau-b test was performed to investigate the correlation between the number of coronary artery stenosis ≥50% and CPET parameters. We analyzed the associations between CFR and CAD by means of logistic regression. Predictor coefficients were estimated by regression models, including the following: (1) only the CFR; (2) only clinical predictors (age, sex, BMI, history of smoking, hypertension, diabetes and hypercholesterolemia); and (3) both the CFR and clinical predictors. The receiver operating characteristic (ROC) curve and the area under ROC (AUC) were used to examine the predictive quality. Medcalc software (Version 20.1.0) was used to compare ROC curves. In addition, we used the category-less net reclassification improvement (NRI) to quantify the degree of correct reclassification when using the model with CRF, compared with the model without CRF [17]. The NRI quantifies the amount of correct change in model-based probabilities introduced by using a model with a new marker. *p* < 0.05 was considered statistical significance.

## 3. Results

### 3.1. Study Participants

A total of 188 male patients and 92 female patients were enrolled in this study. Their details were as follows: age (55.90 ± 8.65 vs. 58.46 ± 7.18 years), body weight (73.02 ± 9.62 vs. 62.32 ± 8.56 kg) and BMI (25.21 ± 2.94 vs. 24.89 ± 3.32 kg/m^2^), respectively (Table 1). There were no major cardiac events during CPET. The spirometry parameters demonstrated a normal response. Further sample baseline characteristics stratified by gender are provided in the Supplementary Materials, Table S2, including the distribution among anamnesis and medication.

### 3.2. CRF Was Correlated with QFR

**Figure 1:**
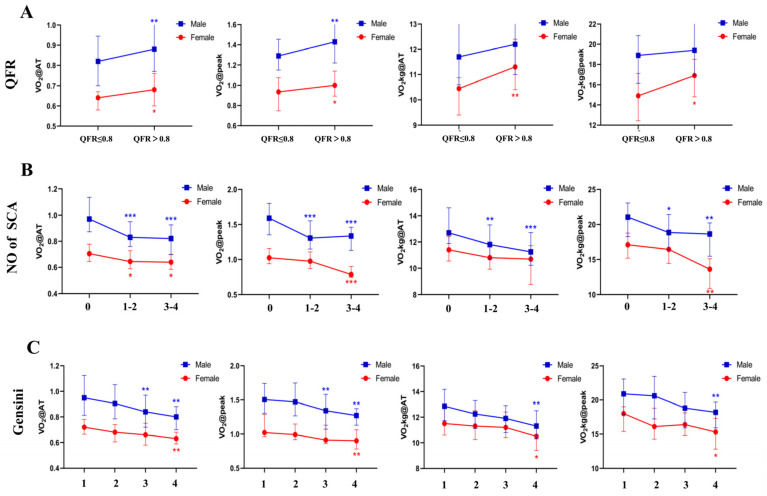
VO_2_@peak, VO_2_@AT, VO_2_kg@peak and VO_2_kg@AT levels according to (**A**) quantitative flow ratio (QFR) (QFR ≤ 0.8 and QFR > 0.8); (**B**) the number of stenotic coronary arteries (SCA) (0, 1–2 and 3–4) and (**C**) Gensini scores (grouped by quartile). The four groups of males were group 1 (Gensini score ≤ 6.0), group 2 (6.0 < Gensini score ≤ 12.5), group 3 (12.5 < Gensini score ≤ 27.5) and group 4 (Gensini score > 27.5), respectively. The four groups of females were group 1 (Gensini score ≤ 3.0), group 2 (3.0 < Gensini score ≤ 7.5), group 3 (7.5 < Gensini score ≤ 14.5) and group 4 (Gensini score > 14.5), respectively. Post-hoc was performed between any two groups for SCA and the Gensini score, while only the significances for the first group were exhibited in the figures. * *p* < 0.05, ** *p* < 0.01, *** *p* < 0.001. VO_2_@peak—peak oxygen uptake; VO_2_@AT—oxygen uptake at anaerobic threshold; VO_2_kg@peak—peak kilogram oxygen uptake; VO_2_kg@AT—kilogram oxygen uptake at anaerobic threshold.

To investigate the relationship between the QFR and CPET, the Spearman test was used, which investigated the correlation between the renumbered QFR and the CPET indices (VO_2_@peak, VO_2_@AT, VO_2_kg@peak and VO_2_@AT). The statistical results may have been affected by the large differences in the gender ratio among the groups. Therefore, all patients were divided into two groups based on their gender (Supplementary Table S3) and the differences were statistically calculated by subgroups. In both the male and female patients, our findings showed that the VO_2_@AT and the VO_2_@peak in the QFR ≤ 0.8 group were significantly lower than they were in the QFR > 0.8 group (Figure 1, Supplementary Table S4). The correlation test that was conducted according to gender indicated that in the male patients the VO_2_@peak (r = 0.176, *p* = 0.016) and the VO_2_@AT (r = 0.161, *p* = 0.027) were correlated with the QFR. In the females, the VO_2_@peak (r = 0.231, *p* = 0.027), the VO_2_@AT (r = 0.212, *p* = 0.043), the VO_2_kg@peak (r = 0.212, *p* = 0.044) and the VO_2_@AT (r = 0.277, *p* = 0.008) were associated with the QFR (Table 2).

### 3.3. CRF Showed Negative Correlation with the Number of Stenotic Coronary Arteries (SCA)

To assess the relationship between the number of SCAs and cardiopulmonary exercise test results, we examined the relationship between the number of SCAs and the reassigned VO_2_@peak, VO_2_@AT, VO_2_kg@peak and VO_2_@AT, using the Kendall’s tau-b test, respectively. We performed categorical analyses to summarize the CPET parameters for three subgroups (Supplementary Table S5). In particular, the median VO_2_@peak and the median VO_2_@AT were highest in the participants with no SCAs, while the median VO_2_@peak and the median VO_2_@AT were lowest in the participants with 3–4 SCAs (Figure 1, Supplementary Table S6). In the male patients, the VO_2_@peak (τ = −0.307, *p* < 0.001), the VO_2_@AT (τ = −0.312, *p* < 0.001), the VO_2_kg@peak (τ = −0.235, *p* < 0.001) and the VO_2_@AT (τ = −0.245, *p* < 0.001) were negatively correlated with the number of SCAs. In the female patients, the VO_2_@peak (τ = −0.230, *p* = 0.01) and the VO_2_@AT (τ = −0.261, *p* = 0.004) were negatively correlated with the number of SCAs. This indicates that the higher the oxygen uptake volume is in CPET, the more likely it is that SCAs exist (Table 3).

### 3.4. CRF Was Correlated with Gensini Score

A Spearman correlation analysis was conducted to preliminarily explore the correlation between the CPET parameters and the Gensini score, which were segmented by gender. We performed categorical analyses to summarize the CPET parameters for four subgroups. Specifically, the VO_2_@AT (male *p* < 0.001, female *p* = 0.006), the VO_2_@peak (male *p* < 0.001, female *p* = 0.011), the VO_2_/kg@AT (male *p* = 0.002, female *p* = 0.031) and the VO_2_/kg@peak (male *p* = 0.001, female *p* = 0.038) were significantly decreased in the four different groups of male and female patients (Figure 1, Supplementary Table S7). The Spearman correlation analysis showed that the Gensini score was highly correlated with the indices of maximal exercise, such as the VO_2_@peak (r = −0.425, *p* < 0.001); the VO_2_ @AT (r = −0.406, *p* < 0.001); the VO_2_kg @AT (r = −0.308, *p* < 0.001); and the VO_2_ kg @peak (r = −0.326, *p* < 0.001) in the male group. The correlation between the Gensini score and the VO_2_@peak (r = −0.338, *p* = 0.001); the VO_2_ @AT (r = −0.368, *p* < 0.001), (r = 0.342, *p* = 0.001); the VO_2_kg @AT (r = −0.259, *p* = 0.013); and the VO_2_kg@peak(r = −0.241, *p* = 0.022) were significantly higher in the female group (Table 4).

### 3.5. The Diagnostic Value and Predictors of the Severity of the Coronary Lesions

**Figure 2:**
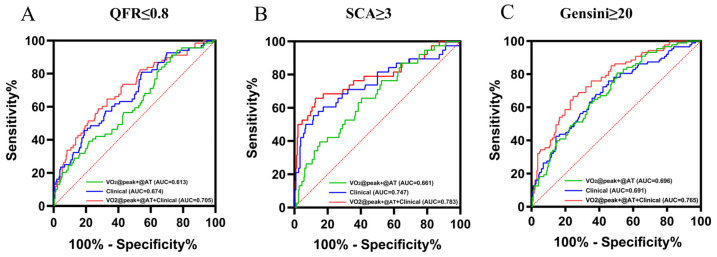
The area under the receiver–operator characteristic (ROC) curves are based on logistic regression models, incorporating conventional risk factors (including age, sex, BMI and history of smoking, hypertension, diabetes and hypercholesterolemia), with and without VO_2_@AT/VO_2prediction_ + VO_2_@peak/VO_2prediction_. AUCs were calculated to distinguish the severe CAD and non-severe CAD patients, measured by quantitative flow ratio (QFR). (**A**): the number of stenotic coronary arteries (SCA), (**B**): the Gensini score, and (**C**): VO_2_@AT—oxygen uptake at anaerobic threshold, and VO_2_@peak—peak oxygen uptake.

In order to reduce the gender interference and combine the similar parameters, we used the VO_2_ predicted value ratio as the independent variable. Figure 2 shows the ROC curves of the VO_2_@AT/VO_2prediction_ + VO_2_@peak/VO_2prediction_ alone; the predictive model of the clinical predictors alone (including age, sex, BMI and the history of smoking, hypertension, diabetes and hypercholesterolemia); and the comprehensive predictive model (clinical predictors and the VO_2_@AT/VO_2prediction_ + VO_2_@peak/VO_2prediction__)_. The AUC of the model, including the VO_2_@AT/VO_2prediction_ + VO_2_@peak/VO_2prediction_ alone, ranged from 0.613 for functionally significant lesions, with QFR ≤ 0.8, 0.661 for SCA ≥ 3, to 0.696 for the Gensini score ≥ 20. The AUC of the clinical model ranged from 0.674 for the QFR ≤ 0.8 and 0.691 for the Gensini score ≥ 20, to 0.747 for SCA ≥ 3. The comprehensive predictor of the VO_2_@AT/VO_2prediction_ + VO_2_@peak/VO_2prediction_ and the clinical model produced a significant increase in the AUC for the QFR ≤ 0.8 (AUC = 0.705), the Gensini score ≥ 20 (AUC = 0.765) and SCA ≥ 3 (AUC = 0.783). The addition of the VO_2_@AT/VO_2prediction_ + VO_2_@peak/VO_2prediction_ to the clinical model produced a significant improvement in the net reclassification, as measured by the NRI in the Gensini score group (11.66%, *p* < 0.01), whereas no significant improvements were observed in the QFR (5.04%, *p* = 0.15) and the SCA groups (9.26%, *p* = 0.09). Regression models with coefficients are provided in the Supplementary Tables S9–S11.

## 4. Discussion

This study aimed to investigate the association between cardiorespiratory fitness, as measured by CPET, and the severity of coronary lesions, comprehensively assessed by the QFR, the number of SCAs and the Gensini score. Well-powered population samples with deeply meticulous measures of CAD are necessary to provide strong evidence for the association between CRF and the severity of coronary lesions. This study demonstrated that CRF is inversely related to the severity of coronary lesions in both male and female patients with CAD (Figure 1). Screening for CRF by CPET provides a strategy for categorizing separate individuals into the categories of severe, mild or moderate CAD.

As reported in the previous literature, CRF is strongly associated with all-cause and cardiovascular mortality [17,18,19] and may be an even better and more important predictor of mortality than traditional risk factors such as hypertension, diabetes, cholesterol levels, and smoking [20]. These studies reported that CRF predicts the degree of coronary atheromatous burden, which is consistent with our observations. Dejana Popovic et al., showed that the ventilation/carbon dioxide production (VE/VCO_2_) and the slope obtained on the treadmill (TM) hold a predictive value in distinguishing between one and two SCAs and three SCAs [18]. Coronary artery severity is difficult to accurately assess by a single criterion, thus we assessed coronary artery severity across the following three aspects: the QFR, the number of SCAs and the Gensini score. Our findings were consistent with the previous study, in which the number of SCAs and the Gensini score were positively associated with the (VE/VCO_2_) slope (Supplementary Table S8). Since age and gender had significant effects on the VO_2_@peak and the other CPET-derived variables, analyzing the entire population would yield inconsistent results. We divided segmented participants into gender groups, thus excluding gender as a major confounder to (theoretically) increase the homogeneity and validity of the results.

It has been reported that PCI with functionally significant lesions (fractional flow ratio [FFR] ≤ 0.8) was associated with improved clinical outcomes in large clinical studies [19]. QFR is a novel method for deriving the FFR. It is mainly based on specific software and simple steps and does not require the use of a pressure wire or the induction of hyperemia [20]. Previous studies have validated that QFR has a high diagnostic accuracy in identifying hemodynamically significant coronary stenosis, with the FFR serving as the reference standard [21]. In line with this expectation, the gender-specific correlation test indicated that low CRF showed a marked correlation with the coronary significant lesions with QFR ≤ 0.8. In addition, we also assessed the relationship between the number of coronary arteries with stenosis ≥ 50% and CRF. Similarly, the results indicated that the lower the oxygen uptake in the CPET, the more likely it is that coronary artery with stenosis ≥ 50% exists. CRF also showed a marked inverse correlation with the Gensini score.

The QFR, the number of SCAs and the modified Gensini scores all demonstrate a strong, direct relationship with CRF. It should be noted that this relationship exists within a CAD population. That is, although several studies have documented that CRF confers risk for CAD versus controls, we describe the ability of CRF to predict an anatomically consequential disease with an adverse prognosis. The plausibility of our results is strengthened by several connecting biological pathways linking CRF and CAD, such as genetical background, metabolic risk and obesity, and physical activity adherence with favorable responses in the cardiovascular system, inflammation, plasma lipids, fat distribution, atherosclerosis and endothelial function [22,23,24]. Moreover, a precise evaluation of the functional consequences of myocardial ischemia before invasive angiography is critical for the success of subsequent revascularization strategies (i.e., PCI or coronary artery bypass grafting [CABG]). Thus, CPET appears to be useful in the quantification of CAD severity and the burden of ischemia. Our study identified the VO_2_@peak/VO_2prediction_ as having a powerful predictive value in distinguishing between one or two SCAs and over three SCAs, QFR ≤ 0.8 and QFR > 0.8, Gensini score < 20 and Gensini score ≥ 20, demonstrating an additional value in the quantification of CAD, using a means other than echocardiography. The CRF markers significantly improved high-risk CAD prediction when added to the conventional risk factors such as age, BMI, sex, smoking and history of hypertension, diabetes and hypercholesterolemia. A combination of CPET and other cardiovascular risk factors can predict severe CAD (Gensini score ≥ 20) more accurately than either can in isolation. Our data suggest that adding the CRF information to the model might lead to an 11.6% net gain with respect to moving the risk estimates toward the correct direction. For patients with low CRF, as defined by the regression model, and who have ischemia and impaired cardiac function caused by severe CAD, a more aggressive form of management for vascular disease may be recommended.

Unlike previous studies that relied on exercise tests to estimate CRF, our study used VO_2_@peak quantification to assess the severity of coronary artery lesions. Although submaximal and maximal exercise testing, as well as non-exercise prediction equations, are deemed feasible and have been shown to have prognostic value, the additional clinical information and the higher external validity due to the superior precision provided by CPET, favors the implementation of directly assessed VO_2_@peak [25]. A study from the FRIEND database, for example, found significant differences between the VO_2_@peak measured directly and the VO_2_@peak estimated from exercise test data, especially at the extremes of CRF [26]. Furthermore, CPET can be used to provide a more accurate interpretation of the measured VO_2_@peak from maximal exercise tests, when compared to previous standards that were based on workload-derived estimations.

More importantly, the evidence suggests that a one-metabolic equivalent (MET) increase in CRF on two maximal exercise tests, separated by an average of 6.3 years, was associated with 15% and 19% reductions in all-cause and cardiovascular disease (CVD) mortality, respectively, among 14,345 men [27]. The evidence supports that better CRF is independently associated with longevity [28]. In another study, a one unit increase in long-term CRF (as measured by the maximal oxygen uptake) was associated with a 7% reduced risk of incident non-fatal myocardial infarction (MI) and a 16% decrease in the risk of incident non-fatal heart failure events [29]. Regardless of an individual’s health status (the traditional CVD risk factors), higher levels of physical activity (PA) and CRF improve the overall CVD risk profile [23]. Key CPET variables hold powerful diagnostic and prognostic utility in patients with cardiovascular disease. CPET also holds considerable promise in gauging the response of a broad range of therapies, including pharmacologic, surgical and lifestyle interventions. The association between the VO_2_@peak and CAD was strong. Therefore, cardiac rehabilitation programs with an exercise training component, such as high intensity interval training (HIIT) and continuous aerobic exercise training (CAET), were found to be safe and could improve the prognosis for CAD patients [30]. Improving CRF, especially the peak VO_2_, may evolve into a primary treatment goal in patients with cardiovascular disease if future randomized trials support this approach.

## 5. Conclusions

The principal and novel finding of our study is that there is a strong, inverse and independent association between the degree of coronary artery disease and CRF, as assessed by standardized CPET. Despite its limitations, the present study has added to the growing body of evidence supporting the potential clinical value of CPET in the diagnosis and assessment of the severity of CAD.

## 6. Limitations

All participants underwent the personalized ramp incremental CPET protocol. It might cause a shorter or longer test phase duration in some participants due to an incorrect decision by the operator. The CPET parameters were easily obtained and interpreted, however, these variables were affected by the training status and were altered in some pathological conditions, including heart failure and ventilatory diseases. Hence, further research in a larger cohort of patients with CAD is warranted to refine the diagnostic and prognostic potential of CPET. Beta blockers and other cardioprotective drugs significantly impact the CPET results and should be discontinued prior to CPET to improve the study’s protocol standardization [31]. There were also some limitations in assessing the severity of coronary artery disease and more accurate evaluation methods still need further exploration. Furthermore, since the use of subgroups reduces the number of patients in each subgroup, a larger sample size is required for further studies.

## Figures and Tables

**Table 1:** Baseline characteristics of study patients.

Variable	Total (*n* = 280)
Age (y)	56.74 ± 8.27
BMI (kg/m^2^)	25.11 ± 3.07
Height (cm)	166.35 ± 7.86
Body weight (kg)	69.59 ± 10.54
**Comorbidities** (*n*, %)	
Myocardial infarction	39 (13.9)
Arrhythmia	41 (14.6)
Hypertension	165 (59.0)
Hyperlipemia	80 (28.6)
Cardiac insufficiency	21 (7.5)
Diabetic mellitus	83 (29.6)
Thyroid dysfunction	16 (5.7)
Noncardiogenic chest pain	77 (27.5)
Cerebrovascular disease	24 (8.6)
**Medications** (*n*, %)	
Aspirin	241 (86.1)
Antiplatelet agents (Ticagrelor or Clopidogrel)	210 (75)
Statins	212 (75.7)
ACEI or ARB	96 (34.3)
CCB	84 (30.0)
β-blocker	171 (61.1)
Nitrates	31 (11.1)
Anti-arrhythmia agent	17 (6.1)
Hypoglycemic drugs or insulin	23 (8.2)
**CPET**	
VO_2_@AT (L/min)	0.79 (0.69, 0.93)
VO_2_@peak (L/min)	1.24 (1.01, 1.47)
VO_2_kg@AT (mL/min/kg)	11.60 (10.70, 13.10)
VO_2_kg@peak (mL/min/kg)	18.20 (15.80, 20.90)
RER@peak	1.18 (1.09, 1.24)
VO_2_@AT/VO_2prediction_ (%)	43.3 (38.2, 50.9)
VO_2_@peak/VO_2prediction_ (%)	67.5 (59.6, 77.2)

Baseline data of subjects. Normal distributed continuous parameters, including age, BMI, height and body weight, were expressed as mean ± SD. Non-normal distributed CPET parameters were expressed as median and interquartile. Categorical variables, including comorbidities and medications, were presented by frequency and percentile. BMI—body mass index, ACEI—angiotensin-converting enzyme inhibitors, ARB—angiotensin receptor blockers, CCB—calcium entry blockers, CPET—cardiopulmonary exercise testing.

**Table 2:** The correlation between oxygen uptake volume and QFR.

CPET Parameters	Male		Female	
	r	*p* Value	r	*p* Value
VO_2_@peak (L/min)	0.176	0.016	0.231	0.027
VO_2_@AT (L/min)	0.161	0.027	0.212	0.043
VO_2_kg@peak (mL/min/kg)	0.094	0.200	0.212	0.044
VO_2_kg@AT (mL/min/kg)	0.067	0.361	0.277	0.008

Spearman test was used to investigate the correlation between the renumbered quantitative flow ratio (QFR) and CPET indices (VO_2_@peak, VO_2_@AT, VO_2_kg@peak and VO_2_@AT). r—spearman correlation coefficient. *p* < 0.05 was considered as statistically significant. VO_2_@peak—peak oxygen uptake; VO_2_@AT—oxygen uptake at anaerobic threshold; VO_2_kg@peak—peak kilogram oxygen uptake; VO_2_kg@AT—kilogram oxygen uptake at anaerobic threshold.

**Table 3:** The correlation between the number of SCAs and CPET indices.

CPET Parameters	Male		Female	
	τ	*p* Value	τ	*p* Value
VO_2_@peak (L/min)	−0.307	0.000	−0.230	0.01
VO_2_@AT (L/min)	−0.312	0.000	−0.261	0.004
VO_2_kg@peak (mL/min/kg)	−0.235	0.000	−0.158	0.08
VO_2_kg@AT (mL/min/kg)	−0.245	0.000	−0.172	0.056

VO_2_@peak, VO_2_@AT, VO_2_kg@peak and VO_2_kg@AT in male and female patients were arranged in descending order and then grouped into 4 groups, respectively. 1 to 4 were arranged into the four groups. Kendall’s tau-b test was used to evaluate the correlation between the number of coronary arteries with stenosis (≥ 50%) and converted CPET indices. τ—Kendall’s tau coefficient. *p* < 0.05 was considered as statistically significant. VO_2_@peak—peak oxygen uptake; VO_2_@AT—oxygen uptake at anaerobic threshold; VO_2_kg@peak—peak kilogram oxygen uptake; VO_2_kg@AT—kilogram oxygen uptake at anaerobic threshold; SCA—stenotic coronary arteries.

**Table 4:** Correlation analyses between CPET parameters and Gensini score.

CPET Parameters	Male (*n* = 188)		Female (*n* = 92)	
	R (Spearman Coefficient)	*p*-Value	R (Spearman Coefficient)	*p*-Value
VO_2_@AT (L/min)	−0.406	0.000	−0.368	0.000
VO_2_/kg@AT (L/min)	−0.308	0.000	−0.259	0.013
VO_2_@peak (mL/min/kg)	−0.425	0.000	−0.338	0.001
VO_2_/kg@peak (mL/min/kg)	−0.326	0.000	−0.241	0.022

Correlation analyses between CPET parameters and Gensini score of males and females, respectively. Correlation analyses between CPET parameters and Gensini score was performed by using Spearman correlation test. *p* < 0.05 indicated statistical significance between CPET parameters and Gensini score. VO_2_@peak—peak oxygen uptake; VO_2_@AT—oxygen uptake at anaerobic threshold; VO_2_kg@peak—peak kilogram oxygen uptake; VO_2_kg@AT—kilogram oxygen uptake at anaerobic threshold.

## Data Availability

The data presented in this study are available on request from the corresponding author. The data are not publicly available due to privacy.

## Supplementary Materials

The following supporting information can be downloaded at: https://www.mdpi.com/article/10.3390/jcm11144170/s1.
