# Supplementary material for: The Value of Cardiopulmonary Exercise Testing in Predicting the Severity of Coronary Artery Disease
Source: J Clin Med. 2022 Jul 18;11(14):4170. doi: 10.3390/jcm11144170 (PMC9320309; doi:10.3390/jcm11144170)
Supplement: Supplementary file 1 [file jcm-11-04170-s001.zip › jcm-1769042-supplementary.pdf]

Supplementary Materials

Supplementary Table S1. Gensini score rule.

Extent of coronary artery stenosis	Lesion site of coronary artery	score	Coefficient
≤25%	LM	1	5
26%-50%	Proximal LAD or LCX	2	2.5
51%-75%	Middle LAD	4	1.5
76%-90%	Distal LAD	8	1
91%-99%	Middle or distal LAD	16	1
100%	RCA	32	1
	subbranch		0.5

Supplementary Table S1. LM: left main coronary artery; LAD: left anterior descending; LCX: left circumflex coronary; RCA: right coronary artery.

Supplementary Table S2

Variable	Male (n=188)	Female (n=92)
Age (y)	55.90 ± 8.65	58.46 ± 7.18
BMI (kg/m^2)	25.21 ± 2.94	24.89 ± 3.32
Height (cm)	170.14 ± 5.72	158.28 ± 5.26
Body weight (kg)	73.02 ± 9.62	62.32 ± 8.56
**Comorbidities** （n, %）		
Myocardial infarction	34 (18.1)	5 (5.4)
Arrhythmia	29 (15.4)	12 (13.0)
Hypertension	109 (57.9)	56 (60.9)
Hyperlipemia	53 (28.2)	27 (29.3)
Cardiac insufficiency	18 (9.6)	3 (3.3)
Diabetic mellitus	57 (30.3)	26 (28.3)
Thyroid dysfunction	9 (4.8)	7 (7.6)
Noncardiogenic chest pain	52 (27.7)	25 (27.2)
Cerebrovascular disease	17 (9.0)	7 (7.6)
**Medications** （n, %）		
Aspirin	168 (89.4)	73 (79.3)
Antiplatelet agents	149 (79.2)	61 (66.3)
(Ticagrelor or Clopidogrel)		
Statins	141 (75.0)	71 (77.2)
ACEI or ARB	66 (35.1)	30 (32.6)
CCB	59 (31.4)	25 (27.2)
β-blocker	123 (65.4)	48 (52.2)
Nitrates	21 (11.2)	10 (10.9)
Anti-arrhythmia agent	14 (7.5)	3 (3.3)
Hypoglycemic drugs or insulin	14 (7.4)	9 (9.8)

Supplementary table S2. Baseline data of male and female subjects were exhibited respectively.

Supplementary Table S3. The number of patients for each QFR group.

QFR	Male	Female	Total
0 (≥1 coronary artery with QFR≤0.8)	53(28.2%)	16(17.4%)	69(24.6%)
1 (no coronary artery with QFR≤0.8)	135(71.8%)	76(82.6%)	211(75.4%)

Supplementary Table S4. Level of VO_2 of the QFR groups

Variable	Male (n=188)			Female (n=92)		
	**QFR≤0.8 (n=53)**	**QFR＞0.8 (n=135)**	***P* value**	**QFR≤0.8 (n=16)**	**QFR＞0.8 (n=76)**	***P* value**
VO_2@AT (L/min)	**0.82** (0.70, 0.95)	**0.88** (0.77, 1.02)	**0.008**	**0.64** (0.58, 0.67)	**0.60** (0.60, 0.76)	**0.039**
VO_2/kg@AT (ml/min/kg)	**11.70** (10.60, 13.35)	**12.20** (11.00, 13.50)	**0.263**	**10.45** (9.40, 10.88)	**11.30** (10.40, 11.30)	**0.009**
VO_2@peak (L/min)	**1.29** (1.15, 1.46)	**1.43** (1.22, 1.67)	**0.006**	**0.94** (0.75, 1.08)	**1.00** (0.89, 1.14)	**0.030**
VO_2/kg@peak (ml/min/kg)	**18.90** (16.15, 20.85)	**19.40** (16.90, 22.30)	**0.154**	**14.90** (12.43, 17.10)	**16.90** (14.80, 18.50)	**0.015**

Supplementary Table S4. The enrolled patients were divided into two groups according to QFR (QFR > 0.8 and QFR≤0.8). CPET parameters were expressed as median (quartiles). Mann-Whitney non-parameter test was used to identify significant difference for each index. $P < 0.05$ was considered statistical significance. VO_2@peak, peak oxygen uptake; VO_2@AT, oxygen uptake at anaerobic threshold; VO_2kg@peak, peak kilogram oxygen uptake; VO_2kg@AT kilogram oxygen uptake at anaerobic threshold.

Supplementary Table S5. The number of patients with SCA.

the number of SCA	Male	Female	Total
0	50(26.6%)	36(39.1%)	86(30.7%)
1-2	108(57.4%)	48(52.2%)	156(55.7%)
3-4	30(15.9%)	8(8.7%)	38(13.6%)

Supplementary Table S5. The number of coronary artery with stenosis ≥50% were recorded for each patient (left main trunk, left anterior descending branch, left circumflex branch, and right coronary artery). The number of male, female patients with 0, 1-2 and 3-4 coronary arteries were shown respectively.

Supplementary Table S6. Level of VO_2 of different number of SCA

Variable	Male (n=188)				Female (n=92)			
	**0 (n=50)**	**1-2 (n=108)**	**3-4 (n=30)**	***P* value**	**0 (n=36)**	**1-2 (n=48)**	**3-4 (n=8)**	***P* value**
VO_2@AT (L/min)	**0.95** (0.81, 1.13)	**0.91** (0.79, 1.05)	**0.84** (0.72, 0.97)	**0.000**	**0.72** (0.67, 0.78)	**0.68** (0.61, 0.74)	**0.66** (0.58, 0.75)	**0.029**
VO_2/kg@AT (ml/min/kg)	**12.85** (11.73, 14.18)	**12.25** (11.13, 13.30)	**11.90** (10.80, 12.90)	**0.000**	**11.50** (10.60, 12.75)	**11.30** (10.25, 12.50)	**11.20** (10.40, 12.40)	**0.271**
VO_2@peak (L/min)	**1.51** (1.30, 1.74)	**1.47** (1.27, 1.75)	**1.34** (1.07, 1.58)	**0.000**	**1.02** (0.96, 1.29)	**0.99** (0.92, 1.15)	**0.91** (0.86, 1.13)	**0.001**
VO_2/kg@peak (ml/min/kg)	**20.9** (17.85, 23.08)	**20.6** (17.23, 23.48)	**18.8** (15.90, 21.10)	**0.002**	**18.0** (15.40, 19.00)	**16.1** (14.25, 18.80)	**16.4** (14.80, 18.10)	**0.009**

Supplementary Table S6. The patients were divided into three groups according to the number of coronary artery stenosis ≥50% (0，1-2，3-4 respectively). CPET parameters were expressed as median (quartiles). Kruskal-Wallis non-parameter test was used to identify significant difference for each index. $P < 0.05$ was considered statistical significance. VO_2@peak, peak oxygen uptake; VO_2@AT, oxygen uptake at anaerobic threshold; VO_2kg@peak, peak kilogram oxygen uptake; VO_2kg@AT kilogram oxygen uptake at anaerobic threshold.

Supplementary Table S7. Level of VO_2 of 4 Gensini score groups

Variable	Male (n=188)					Female (n=92)				
	Group1 (n=48)	Group2 (n=46)	Group3 (n=47)	Group4 (n=47)	*P* value	Group1 (n=25)	Group2 (n=21)	Group3 (n=23)	Group4 (n=23)	*P* value
VO_2@AT (L/min)	**0.95** (0.81, 1.13)	**0.91** (0.79, 1.05)	**0.84** (0.72, 0.97)	**0.80** (0.70, 0.88)	**0.000**	**0.72** (0.67, 0.78)	**0.68** (0.61, 0.74)	**0.66** (0.58, 0.75)	**0.63** (0.59, 0.68)	**0.006**
VO_2/kg@AT (ml/min/kg)	**12.85** (11.73, 14.18)	**12.25** (11.13, 13.30)	**11.90** (10.80, 12.90)	**11.30** (10.30, 12.50)	**0.002**	**11.50** (10.60, 12.75)	**11.30** (10.25, 12.50)	**11.20** (10.40, 12.40)	**10.50** (9.40, 11.50)	**0.031**
VO_2@peak (L/min)	**1.51** (1.30, 1.74)	**1.47** (1.27, 1.75)	**1.34** (1.07, 1.58)	**1.27** (1.13, 1.37)	**0.000**	**1.02** (0.96, 1.29)	**0.99** (0.92, 1.15)	**0.91** (0.86, 1.13)	**0.90** (0.78, 1.06)	**0.011**
VO_2/kg@peak (ml/min/kg)	**20.9** (17.85, 23.08)	**20.6** (17.23, 23.48)	**18.8** (15.90, 21.10)	**18.2** (15.90, 19.70)	**0.001**	**18.0** (15.40, 19.00)	**16.1** (14.25, 18.80)	**16.4** (14.80, 18.10)	**15.3** (12.80, 17.30)	**0.038**

Supplementary Table S7. Gensini scores were grouped by quartile. The four groups of male are group 1 (Gensini score≤6.0), group 2 (6.0 < Gensini score≤12.5), group 3 (12.5 < Gensini score≤27.5) and group 4 (Gensini score > 27.5), respectively. The four groups of female are group 1 (Gensini score≤3.0), group 2 (3.0 < Gensini score≤7.5), group 3 (7.5 < Gensini score≤14.5) and group 4 (Gensini score > 14.5), respectively. Then the enrolled subjects were divided into the four groups according to the Gensini score. CPET parameters were expressed as median (quartiles). Each CPET parameter was compared among the four groups using Kruskal-Wallis non-parameter test to identify significant difference. VO_2@peak, peak oxygen uptake; VO_2@AT, oxygen uptake at anaerobic threshold; VO_2kg@peak, peak kilogram oxygen uptake; VO_2kg@AT kilogram oxygen uptake at anaerobic threshold.

Supplementary Table S8. Correlation between VE/VCO2 slope and QFR, SCA and the Gensini score

	**QFR**		**SCA**		**Gensini score**	
	**r**	***P* value**	***τ***	***p-value***	***r***	***p-value***
VE/VCO_2 slope	-0.006	0.918	0.123	0.006	0.132	0.027

Supplementary table S8. Spearman test was used to examine the correlation between VE/VCO_2 slope and QFR as well as VE/VCO_2 slope and Gensini score. Kendall 's Tau-b test was used to examine the correlation between VE/VCO_2 slope and SCA. $P < 0.05$ was considered as statistically significance.

Supplementary table S9. The logistic regression model of QFR

	Beta coefficient		
	CPET model	Clinical model	CPET +clinical model
VO_2@AT/$VO_{2prediction}$	-0.009	/	-0.015
VO_2@peak/$VO_{2prediction}$	-0.030	/	-0.026
Gender	/	0.374	0.022
Age	/	-0.012	0.006
BMI	/	-0.073	-0.046
Hypertension	/	-0.945	-0.977
Hyperlipemia	/	0.435	0.415
Diabetes	/	-0.638	-0.506
Smoking	/	-1.036	-1.052
Constant	1.291	2.440	3.356

CPET model: VO_2@AT /$VO_{2prediction,}$ VO_2@peak /$VO_{2prediction}$;
Clinical model: gender, age BMI, hypertension, hyperlipemia, diabetes and smoking;
CPET combined with clinical model: VO_2@AT /$VO_{2prediction,}$ VO_2@peak /$VO_{2prediction}$, gender, age BMI, hypertension, hyperlipemia, diabetes and smoking.

Supplementary table S10. The logistic regression model of SCA

	Beta coefficient		
	CPET model	Clinical model	CPET+clinical model
VO_2@AT/$VO_{2prediction}$	0.032	/	-0.398
VO_2@peak/$VO_{2prediction}$	-0.059	/	0.024
Gender	/	-0.160	0.048
Age	/	0.009	-0.547
BMI	/	0.007	0.062
Hypertension	/	-0.516	-0.287
Hyperlipemia	/	0.201	-2.636
Diabetes	/	-0.591	0.042
Smoking	/	-2.473	-0.070
Constant	0.668	-0.113	0.969

CPET model: VO_2@AT /$VO_{2prediction,}$ VO_2@peak /$VO_{2prediction}$;

Clinical model: gender, age BMI, hypertension, hyperlipemia, diabetes and smoking;

CPET combined with clinical model: VO_2@AT /$VO_{2prediction,}$ VO_2@peak /$VO_{2prediction}$, gender, age BMI, hypertension, hyperlipemia, diabetes and smoking.

Supplementary Table S11. The logistic regression model of the Gensini score

	Beta coefficient		
	CPET model	Clinical model	CPET +clinical model
VO_2@AT/$VO_{2prediction}$	-0.007	/	-0.012
VO_2@peak/$VO_{2prediction}$	-0.054	/	-0.055
Gender	/	0.876	0.357
Age	/	0.014	0.044
BMI	/	-0.076	-0.032
Hypertension	/	-0.446	-0.480
Hyperlipemia	/	0.486	0.472

Diabetes	/	-0.381	-0.137
Smoking	/	-1.097	-1.182
Constant	3.153	0.711	2.449

CPET model: VO_2@AT /$VO_{2prediction}$, VO_2@peak /$VO_{2prediction}$;

Clinical model: gender, age BMI, hypertension, hyperlipemia, diabetes and smoking;

CPET combined with clinical model: VO_2@AT /$VO_{2prediction}$, VO_2@peak /$VO_{2prediction}$, gender, age BMI, hypertension, hyperlipemia, diabetes and smoking.
